# Parasitic thyroid nodules: cancer or not?

**DOI:** 10.1530/EDM-14-0027

**Published:** 2014-05-01

**Authors:** Lauren J Baker, Anthony J Gill, Charles Chan, Betty P C Lin, Bronwyn A Crawford

**Affiliations:** 1 Endocrinology DepartmentConcord HospitalSydney, New South WalesAustralia; 2 Pathology DepartmentRoyal North Shore HospitalSydney, New South WalesAustralia; 3 Sydney Medical School, University of SydneySydney, New South WalesAustralia; 4 Anatomical Pathology DepartmentConcord HospitalSydney, New South Wales?Australia

## Abstract

In 2006, a 58-year-old woman presented with thyrotoxicosis. She had undergone left hemithyroidectomy 14 years before for a benign follicular adenoma. Ultrasound imaging demonstrated bilateral cervical lymphadenopathy with enhanced tracer uptake in the left lateral neck on a Technetium-99m uptake scan. Fine-needle aspiration biopsy of a left lateral neck node was insufficient for a cytological diagnosis; however, thyroglobulin (Tg) washings were strongly positive. The clinical suspicion was of functionally active metastatic thyroid cancer in cervical lymph nodes. A completion thyroidectomy and bilateral cervical lymph node dissection were performed. Histology demonstrated benign multinodularity in the right hemithyroid, with bilateral reactive lymphadenopathy and 24 benign hyperplastic thyroid nodules in the left lateral neck that were classified as parasitic thyroid nodules. As there had been a clinical suspicion of thyroid cancer, and the hyperplastic/parasitic thyroid tissue in the neck was extensive, the patient was given ablative radioactive iodine (3.7 GBq). After 2 years, a diagnostic radioactive iodine scan was clear and the serum Tg was undetectable. The patient has now been followed for 7 years with no evidence of recurrence. Archived tissue from a left lateral neck thyroid nodule has recently been analysed for *BRAF* V600E mutation, which was negative.

**Learning points:**

- Thyrotoxicosis due to functional thyroid tissue in the lateral neck is very rare and may be due to metastatic thyroid cancer or benign parasitic thyroid tissue.
- Parasitic thyroid nodules should be considered as a differential diagnosis of lateral neck thyroid deposits, particularly where there is a history of prior thyroid surgery.
- Parasitic thyroid nodules may occur as a result of traumatic rupture or implantation from a follicular adenoma at the time of surgery.
- The use of ablative radioactive iodine may be appropriate, as resection of all parasitic thyroid tissue can prove difficult.
- BRAF mutational analysis of parasitic thyroid tissue may provide extra reassurance in the exclusion of papillary thyroid carcinoma.

## Background

Parasitic thyroid nodules causing thyrotoxicosis are rare; the aetiology and natural history of this diagnosis is unclear. New techniques of mutational analysis of thyroid tissue may provide further prognostic information to guide patient management.

## Case presentation

A 58-year-old woman presented in 2006 with a 3-month history of palpitations and sweating. Thyroid function tests showed tri-iodothyronine (T_3_) toxicosis: thyroid-stimulating hormone (TSH), <0.01 mIU/l (normal 0.3–4.0); free T_3_, 8.0 pmol/l (normal 2.5–6.0) and free thyroxine (T_4_), 17.4 pmol/l (normal 8–22). Anti-thyroglobulin (Tg), anti-microsomal and TSH receptor antibodies were negative. Thyroid function tests one year before were normal.

The patient was previously well and on no regular medication. Her past medical history included a left hemithyroidectomy for a benign follicular adenoma in 1992.

On examination, she was anxious and hypertensive 160/90, but not tachycardic. There were no overt signs of thyrotoxicosis, no palpable goitre and no cervical lymphadenopathy.

## Investigation

Thyroid ultrasound demonstrated a small remnant of the left lobe <1 cm in size and a right thyroid lobe containing small nodules <6 mm in diameter, with specs of calcification. The ultrasound report also noted several grossly abnormal lymph nodes in the left lower cervical area extending laterally (maximum 21 mm diameter); all of these lymph nodes had markedly increased vascular signal. One lymph node in the mid-right cervical area (22 mm diameter) had loss of hilar echoes.

**Figure 1:**
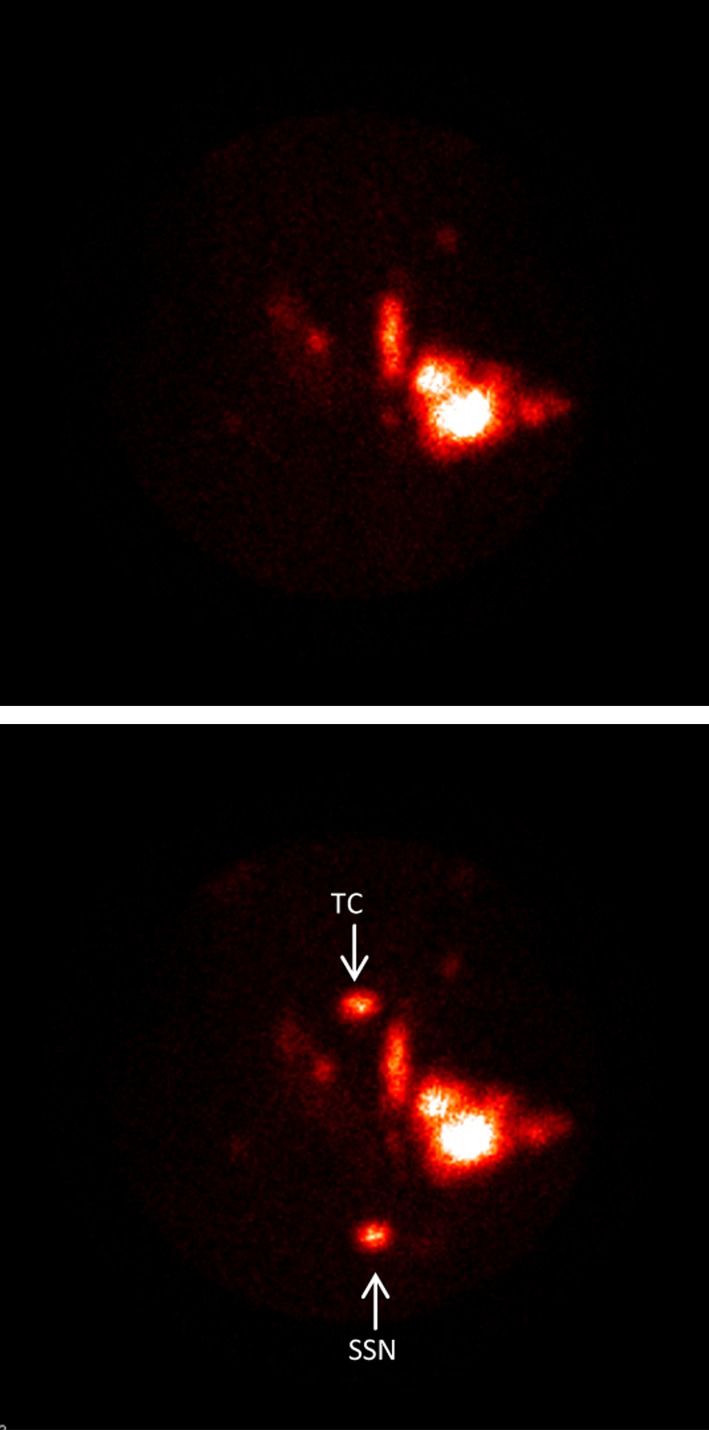
Technetium-99m Thyroid Study. There is almost complete suppression of tracer uptake in the right thyroid lobe. A small amount of uptake is seen in the left thyroid bed area. There is markedly increased tracer uptake in a series of lymph nodes in the left lower neck extending laterally. More superiorly in the left neck, there is uptake in either a remnant of the pyramidal lobe or more abnormal lymph nodes. TC, thyroid cartilage marker; SSN, suprasternal notch marker.

Technetium-99 thyroid scan (Fig. 1) showed almost no uptake in the right thyroid lobe but markedly increased uptake in a number of lesions in the left lower neck extending laterally, which were identified as lymph nodes. There was no uptake in the right side of the neck lateral to the thyroid. The report suggested that this imaging picture was most consistent with hyper-functioning metastatic thyroid tissue within lymph nodes.

Fine-needle aspiration biopsy of bilateral lymph nodes provided insufficient material for cytological diagnosis. Tg washings were performed on the aspirate specimens. The left-sided nodule aspirate had markedly elevated Tg at 13239 μg/l; the right-sided aspirate was negative for Tg.

**Figure 2:**
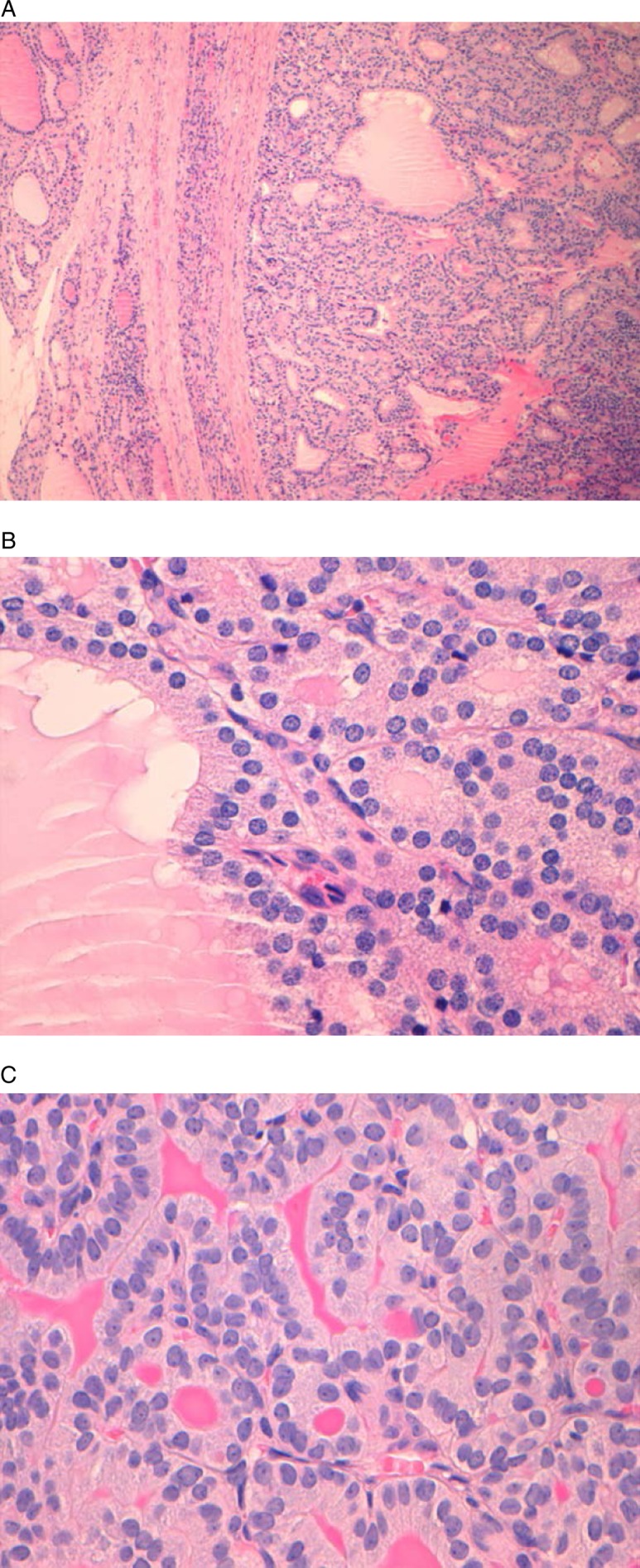
Histopathology from the left hemithyroidectomy (1992). (A) Low power: follicular adenoma measuring 21 mm in diameter is surrounded by a thin and intact capsule. Both the adenoma and the thyroid tissue away from the adenoma show features of hyperplasia (including watery thin colloid and irregularity of the contours of the follicles) consistent with the history of autonomous nodule (H&E original magnification 100×). (B) High power: the follicular cells in the adenoma demonstrate bland cytology being composed of cells with rounded nuclei that lack any atypical cytological features. The presence of watery thin colloid and somewhat columnar cytoplasm is not specific but is seen more commonly in hyperplastic nodules (H&E original magnification 400×). (C) An incidental papillary microcarcinoma, a little <1 mm in maximum dimension, is noted. It is well circumscribed, confined to the thyroid and separated from the resection margin by a narrow soft tissue plane. There is no vascular invasion or perineural spread. The cells lack clear intranuclear pseudo-inclusions, but have crowding, loss of polarity and longitudinal grooves (H&E original magnification 400×).

The positive Tg level in the functioning thyroid tissue in the left side of neck, which appeared to be within lymph nodes, seemed to support a diagnosis of metastatic thyroid cancer. Therefore, the archived pathological slides and paraffin blocks from the left hemithyroidectomy specimen undertaken in 1992 were reviewed. The review confirmed the diagnosis of a benign follicular adenoma measuring 21 mm in diameter with no evidence of capsular penetration or vascular invasion, thus excluding the diagnosis of follicular carcinoma (Fig. 2A and B). On review, the presence of capsular disruption associated with diathermy artefact (suggesting intraoperative rupture of the adenoma) was noted. An incidental papillary carcinoma (encapsulated follicular variant, Fig. 2C) <1 mm in diameter was also noted. This tumour was confined to the thyroid, lacked capsular or vascular invasion and was completely excised. Although the entire thyroid had not been sampled at the time of surgery, and therefore it is impossible to exclude the possibility of other occult microcarcinomas, review of the thyroid away from the adenoma and from the incidental microcarcinoma showed no evidence of malignancy.

## Treatment

The provisional diagnosis was a functional metastatic thyroid cancer from a previously undiagnosed primary cancer. In preparation for surgery, she was rendered euthyroid with carbimazole 10 mg mane. A completion thyroidectomy with central and selective bilateral neck dissection (left, levels II–V and right, levels III and IV) was then performed.

**Figure 3:**
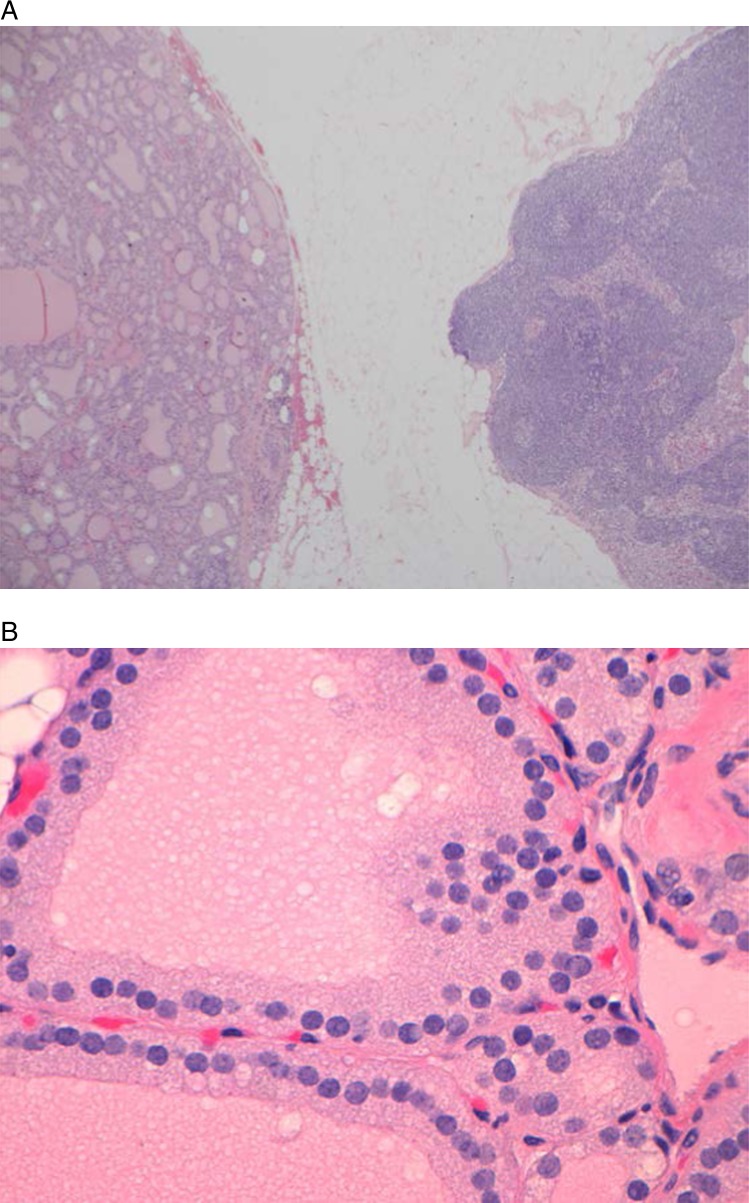
Histology from the completion thyroidectomy and neck dissection. (A) The nodule of thyroid tissue (left) is present in soft tissue and clearly separate from the adjacent benign lymph node (right) from the left lateral neck. There is no histological evidence of invasive growth (H&E original magnification 20×). (B) The cells in the nodule are cytologically bland and again lack nuclear features of papillary carcinoma. Again there are hyperplastic features including watery thin colloid (H&E original magnification 400×).

Histopathology demonstrated a multinodular goitre within the right thyroid lobe with no features of malignancy. In the right neck dissection, all 27 lymph nodes were normal or had non-specific reactive changes. In the left neck dissection, there were 38 normal lymph nodes and 24 nodules (14 in the left paratracheal region, one in left level III and nine in left level IV) of hyperplastic thyroid tissue in soft tissue not associated with lymph nodes (Fig. 3A and B). These nodules showed benign histological features, in particular, there were no nuclear features of papillary carcinoma. Galectin 3, CK19 and HBME1 immunohistochemistry was negative. The nodules were therefore classified as benign parasitic nodules on the presumed basis of intraoperative spillage of the previously resected follicular adenoma.

At 2 months after surgery, she received ablative ^131^I 3.7 GBq dose after T_4_ withdrawal (serum TSH, 123 mIU/l and serum Tg, 1.0 μg/l). The whole-body scan done 3 days after radioactive iodine ablation showed uptake in the thyroid bed only.

## Outcome and follow-up

A follow-up diagnostic whole-body ^123^I scan done 32 months after the completion thyroidectomy, following Thyrogen stimulation (TSH, 223 mIU/l), was clear with no uptake throughout the body and an undetectable Tg level (<0.2 μg/l). The patient has now been followed for 7 years with no evidence of recurrence on ultrasound and with serum Tg remaining undetectable (on thyroxine).

Owing to the clinical suspicion of malignancy and the unknown natural history of parasitic thyroid nodules, archived thyroid tissue was tested for the *BRAF* V600E mutation. This was performed using immunohistochemistry and PCR (followed by sequencing, as well as real-time PCR); all results were negative.

## Discussion

Our diagnosis in this case, taking into account all the clinical and pathological features, is of functional parasitic thyroid nodules in the left lateral neck. Parasitic thyroid nodules are defined as accessory thyroid tissue with no association with lymph nodes, they may or may not be still attached to the thyroid gland proper by a thin fibrous band (1) (2) (3). This is a rare diagnosis and our case is unusual with 24 nodules of functionally active hyperplastic thyroid tissue causing thyrotoxicosis, presenting 14 years after the original thyroid surgery. The natural history of this condition is unclear.

There are two similar cases reported in the recent literature both of which received radioactive iodine ablation post-operatively (4) (5). The first case (4) previously reported was of a 17-year-old woman who underwent a subtotal thyroidectomy for Graves' disease. She developed recurrent thyroid nodules 4 years after initial surgery in the subcutaneous tissue around her surgical scar, the suprasternal notch and laterally on both sides of the neck. This was managed surgically. She had further nodular recurrence over the next 2 years that was managed with two doses of radioactive iodine and suppressive T_4_. Histology demonstrated hyperplasia of the thyroid gland with a follicular adenoma. The second reported case (5) is of a 75-year-old woman who presented 18 years after a total thyroidectomy for benign goitre with recurrent thyroid tissue in the right submandibular area This was managed with resection and radioactive iodine to ablate any residual thyroid tissue.

The differential diagnosis of our patient's presentation includes: seeding from prior thyroid surgery, thyroid rests of embryological origin and metastatic thyroid cancer despite bland histology. Seeding of thyroid tissue from surgery usually results in either multiple subcutaneous thyroid nodules or thyroid tissue surrounded by connective tissue in the field of a previous operation (4). ‘Thyroidosis’ may be the preferred terminology in this instance as this term recognises that the pathophysiology represents a similar phenomenon to ‘splenosis’ in which spillage of splenic cells following trauma or surgery results in functional implants of splenic tissue disseminated within the abdomen or pelvis. Similar to this, but less well recognised, is ‘parathyromatosis’ in which benign parathyroid tissue spills and seeds at surgery, causing recurrent and persistent hyperparathyroidism (6). In our case, however, the lateral neck thyroid nodules were not within the operative field of the original left hemithyroidectomy which did not include any neck dissection in 1992. The hyperplastic thyroid nodules were in an anatomical distribution that was consistent with paratracheal and lateral neck lymph nodes. The histology of the hyperplastic thyroid neck nodules did, however, resemble that of the hyperplastic follicular adenoma from the hemithyroidectomy in 1992.

Thyroid rests from embryological origin occur in over 50% of patients (7). They are often bilateral, occurring most commonly in the midline, along the line of the thyrothymic tract (7). In our case, it would be unlikely that such extensive laterally placed thyroid nodules would be from embryological origin. In addition, there are no reports of embryological rests developing autonomous function although it is possible that pre-existing rests of thyroid tissue of embryological origin could undergo hyperplasia if exposed to similar stimulatory intracellular mechanisms that lead to the development of an intrathyroidal follicular adenoma.

Metastatic thyroid cancer despite bland histology was also considered a potential differential diagnosis. In this case, there was no association of the hyperplastic nodules with lymph node tissue. Re-examination of the left hemithyroidectomy specimen from 1992 confirmed the previous diagnosis of a follicular adenoma with no malignant features. A papillary thyroid microcarcinoma was also found; however, the parasitic thyroid nodules in our case had no nuclear features of papillary thyroid carcinoma. Given that the pre-surgical provisional diagnosis was of metastatic thyroid cancer, despite the lack of subsequent histological confirmation of malignancy, our patient was managed with radioactive iodine ablation due to the clinical suspicion of malignancy and the extensive and functionally active parasitic thyroid nodules.

Molecular markers may be useful in this situation as a guide to the malignant potential of lateral thyroid tissue (8). The use of *BRAF* mutational analysis has been described as a promising diagnostic and prognostic indicator of papillary thyroid carcinomas (9). *BRAF* V600E is an activating mutation that encodes B-Raf, a serine/threonine protein kinase that is involved in directing cell growth (8). The incidence of *BRAF* V600E mutation in papillary thyroid carcinoma is between 32 and 73% (8); it is specific for papillary, poorly differentiated and anaplastic thyroid carcinomas (10). A recent meta-analysis has shown that BRAF is significantly associated with recurrence, lymph node metastasis, extra-thyroidal extension and advanced stage (10). In our case, the negative predictive value of the BRAF analysis was reassuring.

On balance, we feel that the most likely mechanism behind the development of the functional lateral thyroid nodules seen in this case is thyroidosis due to seeding from the previous thyroid surgery. Our case resembles the two previously reported cases where thyroid tissue was found at sites outside of the operative field (lateral neck (4) and submandibular region (5)). Thyroid cells, spilt at the time of the original surgery, may be able to seed the lateral neck through lymphatic channels or other manners of transport yet to be determined.

## Patient consent

See attached.

## Author contribution statement

L Baker was responsible for the case report and literature review. B Crawford edited and finalised the case report, and is the treating physician. A Gill, C Chan and B Lin assisted with pathology slides and discussion.
